# Clinical and genetic analysis of children with glucose transporter type 1 deficiency syndrome

**DOI:** 10.3892/mi.2024.181

**Published:** 2024-07-19

**Authors:** Hao Qian, Guohuan Ying, Haifeng Xu, Shangyu Wang, Bing Wu, Xin Wang, Hongdan Qi, Mingying He, M. Jalal Ud Din, Tingting Huang, Yimei Wu, Gang Zhang

**Affiliations:** Department of Neurology, Children's Hospital of Nanjing Medical University, Nanjing, Jiangsu 210006, P.R. China

**Keywords:** glucose transporter type 1 deficiency syndrome, children, solute carrier family 2 member 1, ketogenic diet, epilepsy

## Abstract

Glucose transporter type 1 deficiency syndrome (GLUT1-DS) is a rare metabolic encephalopathy with a wide variety of clinical phenotypes. In the present study, 15 patients diagnosed with GLUT1-DS were selected, all of whom had obvious clinical manifestations and complete genetic testing. Their clinical data and genetic reports were collated. All patients were provided with a ketogenic diet (KD) and an improvement in their symptoms was observed during a follow-up period of up to 1 year. The results revealed that the 15 cases had clinical symptoms, such as convulsions or dyskinesia. Although none had a cerebrospinal fluid/glucose ratio <0.4, the genetic report revealed that all had the solute carrier family 2 member 1 gene variant, and their clinical symptoms basically improved following the use of the KD. GLUT1-DS is a genetic metabolic disease that causes a series of neurological symptoms due to glucose metabolism disorders in the brain. Low glucose levels in cerebrospinal fluid and genetic testing are key diagnostic criteria, and the KD is a highly effective treatment option. By summarizing and analyzing patients with GLUT1-DS, summarizing clinical characteristics and expanding their gene profile, the findings of the present study may be of clinical significance for the early recognition and diagnosis of the disease, so as to conduct early treatment and shorten the duration of brain energy deficiency. This is of utmost importance for improving the prognosis and quality of life of affected children.

## Introduction

The human brain comprises a mere 2% of the weight of an adult individual; however, it requires a full 25% of the energy consumed by the individual, and the brain of a child requires 3-fold the amount of energy as does the brain of an adult (1). Glucose, being the main energy source in biological material, is also the major energy source in the central nervous system; yet, it cannot pass through the lipid bilayer structure of the cell membrane into cells freely, and must traverse the blood-brain barrier (BBB) via primarily, the glucose transporter 1 (GLUT1) to deliver glucose to the brain (2). The level of GLUT1 can consequently affect brain function and the general health of an individual (3).

GLUT1 deficiency syndrome (GLUT1-DS) is a rare metabolic disease of the nervous system, first reported by De Vivo *et al* in 1991(4). The current incidence is ~1:83,000(5) and the majority of cases are sporadic. It mainly occurs due to mutations in the solute carrier family 2 member 1 (*SLC2A1*) gene, resulting in the reduced or partial loss of GLUT1 expression; thus, glucose cannot effectively pass through the BBB, leading to neuronal energy deficiency, impaired brain function, and finally, to a series of neurological symptoms (6). Low glucose levels have been found in the cerebrospinal fluid (CSF) of these patients. A low CSF/blood glucose ratio (<0.4) is one of the most critical parameters in the diagnosis of GLUT1-DS (7,8). Furthermore, the diagnosis can be genetically confirmed if a pathogenic variant is detected in the *SLC2A1* gene (9). Therefore, the diagnosis can be made following the detection of a pathogenic variant in *SLC2A1* or the CSF criteria either alone, or in combination (10). The disease is classically characterized by low intelligence, microcephaly, seizures and movement disorders. According to the clinical phenotype of children, the disease can be divided into classic and non-classic GLUT1-DS. The onset of GLUT1-DS is early and the symptoms are severe. The main manifestations of chronic encephalopathy are infantile drug-resistant epilepsy, developmental delay, acquired microcephaly, mixed dyskinesia with ataxia and dystonia. The onset of atypical GLUTl-DS occurs at a relatively late stage and mainly presents with mild to moderate clinical symptoms, such as episodic dystonia, migraines and atypical childhood absence episodes. The ketogenic diet (KD), which has been shown to provide more ketone bodies as an alternate source of energy for brain metabolism, is currently the most effective treatment (11). The KD refers to a high-fat, low-carbohydrate diet, in which more fat is metabolized to produce ketone bodies that cross the BBB via monocarboxylate transporters to replace glucose as energy for the brain.

The present study describes the clinical manifestations, genetic testing, treatment and prognosis of 15 patients with GLUT1-DS diagnosed at the authors' hospital.

## Patients and methods

### Study participants

A total of 15 patients diagnosed with GLUT1-DS at the Department of Neurology of Children's Hospital of Nanjing Medical University (Nanjing, China) were included in the present retrospective cohort study. The inclusion criteria were as follows: i) Children diagnosed with GLUT1-DS in the Neurology Department of the hospital, meeting the latest 2020 diagnostic criteria as outlined by the ‘Glut1 Deficiency Syndrome (Glut1DS): State of the art in 2020 and recommendations of the international Glut1DS study group’ (7); ii) children who underwent advanced genetic testing revealing an *SLC2A1* mutation; iii) children with comprehensive clinical data; iv) Children whose parents/guardians volunteered for them to participate in the study, demonstrated good compliance and were cooperative during follow-up. The exclusion criteria were as follows: i) Poor family compliance, uncooperative patients and children with missing clinical data; ii) clearly an acquired brain disease accompanied by corresponding structural abnormalities or the existence of immune, infectious and organic causes.

The present study included 15 patients and they were monitored for up to 1 year. Extensive clinical data, including sex, age at onset, age at diagnosis, clinical manifestations, blood glucose levels, CSF glucose levels, imaging examination results, electroencephalography (EEG) results, genetic test results and disease remission were collected (Table I, Table II and Table III). As regards the treatment response, the anti-seizure drugs and treatment regimens used in the overall treatment of all children are summarized in Table IV. Moreover, the mutation sites were predicted by PyMol Viewer (https://pymol.org/) and SWISS-MODEL (https://swissmodel.expasy.org/). The residues of the missense mutation site and the nearby functional sites were illustrated using PyMol Viewer (the *SLC2A1* gene homologous protein model (id: 6THA) was established in the SWISS-MODEL, and the homologous protein was then imported into PyMol Viewer for visual molecular docking).

The present study involving human participants was reviewed and approved by the Ethics Committee of the Children's Hospital of Nanjing Medical University. Written informed consent to participate in the study was provided by the legal guardian/next of kin of each participant.

### Patient data

Clinical data, neuroimaging studies and the results of EEG were obtained from medical records and the examination of the patients during clinical visits. The patients underwent a lumbar puncture in a fasting state (following 4-6 h of fasting) as part of the initial enrollment in the study. Blood samples for glucose measurements were obtained prior to the lumbar procedure to avoid stress-related hyperglycemia. The genomic DNA of the patients and their parents was extracted from EDTA blood samples according to standard procedures and was carried out by Chigene Genetics. The parents of the patients provided written informed consent.

## Results

### Clinical characteristics

Of the 15 included patients, 7 were males and 8 were females. Their average age was 4 years and 11 months (range, 1 year and 8 months to 12 years and 6 months). The average age at seizure onset was 27.71 months (range, 12 to 57 months).

The clinical features, seizure types and neuroimaging features of the patients are summarized in Table I. Of these 15 children, 10 had seizures. The seizures were of mixed types, including 8 cases of generalized seizure, the most common seizure type, and 2 cases of focal seizure. Of the 15 cases, 9 were classified as classical and 6 as non-classical GLUT1-DS. In total, 11 children were treated with anti-epileptic medications prior to KD therapy; however, the medications had a minimal effect. Movement disorders were reported in 6 patients. Developmental delay was observed in 14 patients, and none of the children had microcephaly. Case nos. 6 and case 13 presented with confusion at the beginning of the disease. Of note, case nos. 6 and 13 (shown in Table I) exhibited frequent convulsions accompanied by changes in consciousness in a short period of time and were admitted to the pediatric intensive care unit of the hospital. Following active treatment, their condition improved.

### Biochemical characteristics and neuroimaging results

A fasting lumber puncture was performed in 9 patients, CSF was analyzed and a low CSF glucose level at 1.34-2.36 mmol/l (average, 1.85 mmol/l), combined with a low CSF/blood glucose ratio of 0.33-0.51 were observed. All patients underwent brain magnetic resonance imaging (MRI) and 13 patients underwent EEG. Only 9 cases exhibited abnormalities on the brain MRI, such as arachnoid cyst, ventricle plump, and hypomyelination and abnormal signals in the right frontal lobe. The EEG revealed generalized epileptiform discharges or background slowing in 8 cases. No abnormalities were found in the remaining 5 cases. These data are summarized in Table II. Biochemical and ultrasound results were also obtained. Each child underwent a cardiac examination and no obvious murmurs were heard. No obvious abnormalities were found on the cardiac ultrasound, electrocardiography and myocardial enzyme spectrum. No notable liver and kidney abnormalities were found in combination with the biochemical and ultrasound results.

### Molecular genetic analysis

All patients underwent genetic testing and were found to carry pathogenic mutations in the *SLC2A1* gene, including 10 patients with missense, 2 patients with frameshift mutations and 3 patients with splice-site mutations (Table III).

A missense variant [chr1:43395436, NM_006516.2: c.695(exon6)G>A, p.Arg232His] in the *SLC2A1* gene was found in case no. 4, which originated from his father, although his father did not have the phenotype. This mutation was previously reported in a patient with absent epilepsy (12), but not in the GLUT1-DS patient population, at least to the best of our knowledge, and it was defined as likely pathogenic. However, according to the Human Gene Mutation Database (HGMD; http://www.hgmd.cf.ac.uk/ac/index.php), it shared the same missense mutation or non-frame-shifting mutation as the identified pathogenic mutation, or shared the same amino acid changes. Combined with the clinical manifestations in this child, it was considered that this gene variant is related to the phenotype of patients, and is a new mutation that has not been previously reported.

A total of 15 site mutations were detected in these children, of which nine mutations have been previously reported (13-17). In addition to case no. 4, the mutation sites in case nos. 6, 7, 11, 12 and 15 have not been previously reported, at least to the best of our knowledge. In patient no. 6, we found a missense variant [chr1:43396415, NM_006516.2: c.398(exon4)G>A, p.Cys133Tyr] in the *SLC2A1* gene, which was described as likely pathogenic, while this missense variation was located in the pathogenic hot spot (there are more than three harmful mutations within the range of ~10 bp), and case no. 6 had clinical manifestations, such as convulsions, developmental lag and a markedly decreased glucose content in CSF. Therefore, it was concluded that the mutation is consistent with the phenotype and is a new, previously unidentified mutation in exon 4.

In case no. 7, two site mutations were found in the *SLC2A1* gene, namely chr1:43424514, NM_006516.2: c.275+2(IVS3)T>G and chr1:43424514, NM_006516.2: c.192(exon4)G>C; in fact, the former was defined as a pathogenic mutation as the loss of function (LOF) mutation led to a possible loss of gene function, while the base change in the latter did not affect the transcription of amino acids and generally did not lead to this disease.

**Figure 1:**
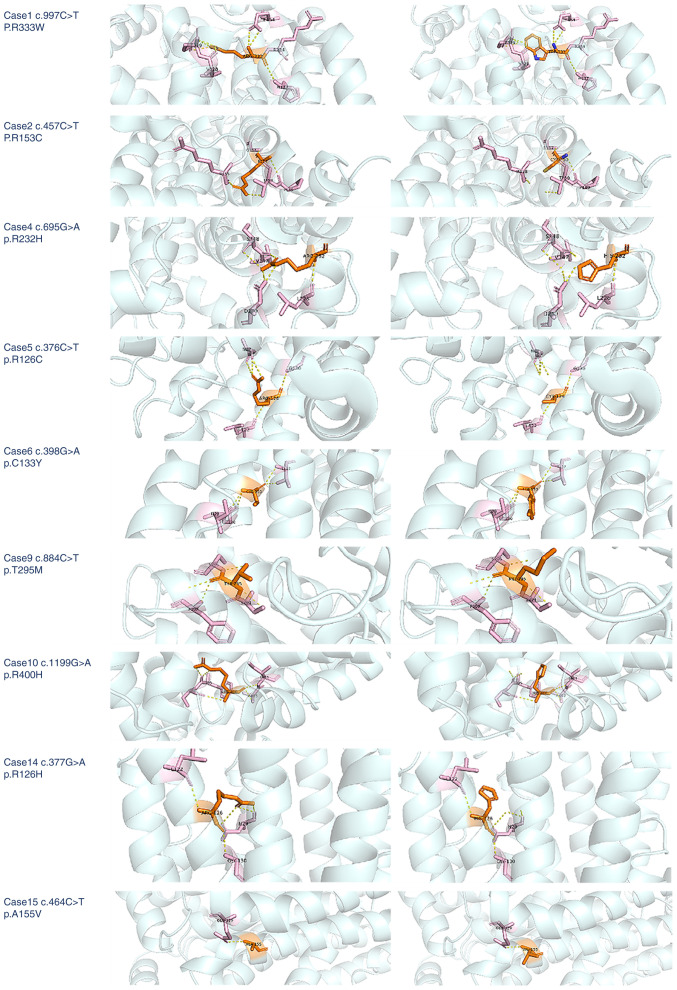
Structural analysis of WT and the variant *SLC2A1* with mutations. The residues of missense mutant sites together with the nearby functional site are illustrated in WT and variant SLC2A1 using PyMol Viewer. The computed hydrogen bonds are shown as yellow dashed lines. Residues of the mutant sites are highlighted in orange solid lines. WT, wild-type.

**Figure 2:**
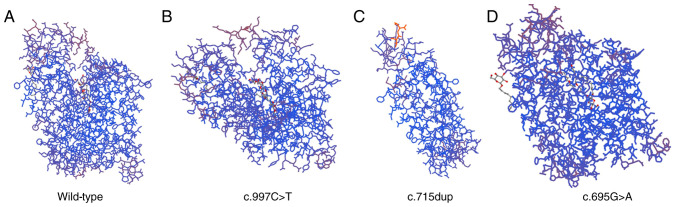
Structural analysis of wild-type and the variant *SLC2A1* with missense mutations and frameshift mutation. Protein prediction software (SWISS-MODEL) was used to predict the three-dimensional structure of proteins. (A) Wild-type, (B) case no. 1, (C) case no. 3, (D) case no. 4. (B-D) Case nos. 1, 3 and 4 were mutant.

Gene sequencing maps were representatively selected for case nos. 4, 5, 6 and 7. The residues of the missense mutation site and the nearby functional sites were illustrated using PyMol Viewer (https://pymol.org/); the *SLC2A1* gene homologous protein model (id: 6THA) was established in the SWISS-MODEL (https://swissmodel.expasy.org/), and the homologous protein was then imported into PyMol Viewer for visual molecular docking. The mutations may affect the protein structure by altering hydrogen bonding and spatial conformation (Fig. 1). The protein prediction software SWISS-MODEL (6THA: X-ray Diffraction, 2.40 Å. ‘Crystal structure of human sugar transporter GLUT1 (SLC2A1) in the inward conformation’; Released 2020-11-25) was used to predict the three-dimensional structure of proteins caused by missense/frameshift mutations in addition to the splice site mutation in case no. 7. It was predicted that c.997C>T in case 1, c.695G>A missense mutations in case no. 4, and c.715dup frameshift mutation in case no. 3 affected the protein structure (Fig. 2). However, missense mutations in case nos. 2, 5 and 6 did not cause changes in protein structure.

### Response to treatment

In the early stages of the disease, the children with convulsions were treated with anti-epileptic therapy, and almost all received various drugs, as shown in Table IV, had a minimal effect in controlling the seizures. Therefore, when they were diagnosed with GLUT1-DS, they received the KD and were followed-up for 1 year. The results revealed that case nos. 1, 2, 3, 6, 8, 9, 11 and 14 had no recurrent convulsions, while case nos. 7 and 15, who had convulsions as the first symptom, still had occasional convulsions due to a short period on the KD, but had fewer attacks than previously. In addition, the 6 children with mobility disorders as their first symptom exhibited an improvement in motor instability after the KD. The main symptom in case no. 4 was delayed speech and motor development; however, when he started KD therapy, his motor skills markedly improved and he can now walk, say single words and call out to individuals. Dyspraxia in case no. 5 also markedly improved. The KD emphasizes the proportion of fat in the diet, and the proportion commonly used in clinical practice ranges from 2:1 to 4:1.

Blood glucose and blood ketones were monitored throughout the period of KD treatment, and there were no severe side-effects at any stage of the KD, and none of the patients dropped out of the study.

## Discussion

GLUT1 is the earliest identified GLUT protein, and contains 492 amino acids and 12 alpha-helical stranded transmembrane proteins. The sequence is highly conserved, and the known human GLUT1 protein has 98% homology with the amino acid sequence of rat, mouse, rabbit and pig (18).

*SLC2A1*, is the only gene related to GLUT1-DS, and Mueckler *et al* (19) successfully cloned it from the cDNA library of the human hepatoma cell line for the first time in 1985, which contains 10 exons and 9 introns. The *SLC2A1* gene mutation is the main cause of GLUT1-DS, and this has been found in >90% of reported cases (20), which is basically a heterozygous mutation, including missense mutation, transcoding mutation, nonsense mutation, splicing site mutation, large fragment deletion and interposition (21). All 15 patients in the present study had the *SLC2A1* gene mutation, 66.7% of which were missense mutations.

The clinical manifestations of GLUT1-DS vary and can be divided into three types. Type I is typical, accounting for ~85% of all cases, the main manifestations of which are mental retardation and epilepsy, with or without motor disorders. Type II is mainly manifested by low intelligence, dyskinesia, but without epilepsy; Type III, is characterized by paroxysmal movement-induced dyskinesia, with or without epilepsy, and the latter two types are also known as atypia, accounting for ~15% of cases.

Epilepsy is one of the most common clinical symptoms of GLUT1-DS, and ~90% of patients have different forms of seizures. Generalized epileptiform spike-wave patterns are the most common. Hewson *et al* (22) reviewed the seizure patterns of 99 patients with GLUT-DS, of whom 89% had extensive spikes or spike-wave electrical activity on EEG. Of all seizure types, generalized tonic-clonic seizures are the most common, and other heterogeneous seizure types, including absence, myoclonic and tonic-clonic seizures have also been reported (23). In addition, some studies have found that the form of EEG was age-dependent. Children <2 years of age are more likely to exhibited focal characteristics on the EEG, and a trend in focal slowing down or epileptic discharge can be observed (24). Nonetheless, in the present study, 10 of the 15 children had seizures, and most were generalized, which is consistent with other studies (25,26). Due to mutation of the *SLC2A1* gene, the function of GLUTl is impaired, the glucose level in the central nervous system is reduced, and the energy supply is insufficient, resulting in abnormal changes in nerve excitability. Therefore, LOF mutation caused by *SLC2A1* gene mutation is associated with the occurrence of epilepsy and other symptoms in patients with GLUT1-DS.

GLUT1-DS has also been shown to be associated with a variety of movement disorders, including, but not limited to choreoathetosis, dystonia, ataxia and alternating hemiplegia. Ataxia is one of the most common movement disorders, but is usually chronic and occurs with other classic symptoms (27). The retrospective study by Pons *et al* (28) also confirmed this point. They observed the clinical manifestations of 57 patients with GLUT1-DS and found that 89% were accompanied by gait disorders, with ataxic-spasm and ataxia being the most common, 86% of them had limb dystonia, 75% had mild chorea, 16% had myoclonus, 21% had dyskinesia and 28% had a non-epileptic emergency, including ataxia, weakness, Parkinson's disease and non-motor dyskinesia (28). Ramm-Pettersen *et al* (29) demonstrated that these movement disorders reflect chronic nutrient deficiencies during brain development, which may be alleviated by a long-term use of the KD. In the present study, there were 6 children with motor disorders, mainly manifested as paroxysmal lower limb weakness and were prone to falls, which were improved by the KD. In the 15 patients in the present study, the authors attempted to analyze the association between the treatment response and mutation pattern. Due to the small sample size, no differences were found. When reviewing previous studies (6,29-34), no significant differences were found between the treatment response and mutation pattern. This may be related to the degree of damage to the gene itself.

The present study also found that all 15 children had varying degrees of language motor development lag, which is consistent with known research results, as the developing brain in patients with GLUT1-DS is unable to obtain sufficient energy for its growth from infancy, leading to cerebral dysfunction (35). As shown in a previous study involving 181 patients with GLUT1-DS, ~69.7% of neurodevelopmental abnormalities were closely related to the incidence of epilepsy, and in epileptic patients, 75.6% had varying degrees of mental motor lag (35). However, due to the small number of cases included herein, the authors were unable to confirm the association between developmental status and the incidence of epilepsy.

For the diagnosis of GLUT1-DS, CSF/blood glucose ratio and gene testing are critical. Among the 15 children in the present study, the CSF/glucose ratio fluctuated between 0.33 and 0.51, and 4 of the 9 children had ratio results >0.4, which lacked high sensitivity and specificity. Combined with the diagnostic criteria for children (07), the absolute decrease in CSF glucose level is more reliable than the decrease in CSF glucose/glucose ratio in the clinic. All the children in the present study had completed genetic testing, and *SLC2A1* gene mutations were found. However, in recent years, the 3-O-methyl-D-Glucose (3-OMG) uptake assay has also emerged as a new test (14). GLUT1 protein is not only widely expressed in brain capillary endothelial cells, astrocytes, neurons and microglia, but is also highly expressed on erythrocyte membranes, and is a key carrier protein of glucose 3-OMG into erythrocytes; therefore, the 3-OMG uptake assay can be used to evaluate the function of GLUT1. When the quantity of GLUT1 protein is reduced or its function is lost, the ability of red blood cells to take up glucose decreases. Yang *et al* (36) studied 109 patients with suspected GLUT1-DS and found that gene mutations were detected in 70 of 74 patients with a reduced glucose intake, compared with only 1 in 35 patients with a normal glucose intake. By inference (36), the majority of patients with GLUT1-DS had a decreased erythrocyte glucose uptake, and when they increased the erythrocyte glucose uptake rate from 60 to 70% of the control group, the sensitivity and specificity reached 99 and 100%, which has resulted in the 3-OMG uptake assay being a critical complementary means of genetic testing and has great significance in confirming GLUT1-DS. However, the 3-OMG uptake assay requires fresh red blood cells with metabolic function; therefore, transporting samples over a long distance will be a key issue in promoting this assay.

The findings of the present study demonstrated that regardless of the initial symptoms, there was an improvement after the KD, which is a high-fat, low-carbohydrate diet. The KD was effective in all 15 children with epilepsy, movement disorders and cognitive improvement, and these symptoms improved within 3 months following the initiation of the KD. The KD can replace glucose with ketone bodies as fuel for the brain, preventing seizures, movement disorders and cognitive impairment, and the earlier it is commenced, the greater its effectiveness (37,38).

Leen *et al* (39) conducted a study on 57 patients with GLUT1-DS, and found that the KD was effective in 86% of the patients with epilepsy, and significantly reduced movement disorder in 48% of the patients with classical phenotype and 71% of the patients with non-classical phenotype. Ramm-Pettersen (29) demonstrated following the long-term observation of patients with GLUT1-DS, that those who were diagnosed and started the KD within a few months after birth had better psychomotor development, while those who commenced the KD later significantly lagged behind their peers. This conclusion has also been verified by other studies (11,22).

In a previous study, researchers from several hospitals in China have retrospectively collected clinical data from 19 children with GLUT1-DS; all patients were treated with the KD, which was effective in 18 cases in terms of seizure control, 11 cases had dyskinesia improvement and 18 cases had cognitive function improvement (40). In the present study, it was found that the KD was effective in improving children's symptoms. This is consistent with the findings of previous studies both nationally and internationally (29,40).

The traditional KD has a strict energy ratio, maintaining the blood ketone body level in the range of 3-4 mg/dl. While maintaining that level of ketone bodies, the body is prone to fatigue, diarrhea and other side-effects, particularly in adult patients, and can reduce the compliance of patients. The Modified Atkins Diet (MAD) can maintain blood ketone levels, the therapeutic effect is similar to KD, and it is better tastewise (41). In previous studies, among patients with GLUT1-DS who were treated with the MAD, the frequency of seizures was reduced by 70-90, and >50% of patients exhibited an improvement in persistent motor disorder symptoms, such as paroxysmal dyskinesia or ataxia (41-43). The MAD was effective for GLUT1-DS, and could further reduce the fat content in the dietary ratio, which has long-term significance for improving food taste and reducing the adverse reactions associated with the high ester diet, and can help improve the compliance of patients with GLUT1-DS.

On the other hand, GLUT1-DS is mainly due to the deficiency of GLUT1 protein expression, which causes metabolic disorders in the brain; thus, gene replacement will become a new therapeutic direction. Nakamura *et al* (44) generated an adenovirus vector aAV9/3 in which human *SLC2A1*-myc-DDK was expressed under the human GLUT1 promoter (AAV-GLUT1), that was intracerebroventricularly injected into GLUT1-deficient mice, and the distribution of exogenous GLUT1 protein in the brain and other organs was analyzed. Their results revealed that exogenous GLUT1 protein was strongly expressed in the cerebral cortex, hippocampus and thalamus, while motor function and CSF glucose levels were significantly improved. Their study provides a basis for gene-level therapy of GLUT1-DS in the future (44).

The present study had certain limitations, which should be mentioned. These include the small sample size and only a 1-year follow-up time. In the future, the authors' research group aims to further expand the collection of cases.

In conclusion, GLUT1-DS is a genetic metabolic disease that causes a series of neurological symptoms due to glucose metabolism disorders in the brain. The decrease in the CSF/blood glucose ratio in CSF is a key indicator in the diagnosis of this disease. The diagnosis in the majority of cases can be confirmed by *SLC2A1* gene testing. GLUT1-DS is a genetic disorder and the most effective treatment is currently the KD. However, the disease is difficult to diagnose given its clinical features, with the mean time to diagnosis in the present study cohort being 4 years and 8 months. Therefore, by reporting relevant cases, this will publicize the disease more and enrich the gene pool, and will provide the relevant basis for earlier diagnosis. Early diagnosis can result in early treatment, which will reduce the duration of nervous system energy deficiency in order to improve the prognosis of affected children.

## Figures and Tables

**Table I:** Clinical data of the patients with GLUT1-DS in the present study.

				Clinical features					
Patient no.	Sex	Current age	Age at onset	Microcephaly	MD	DD	Seizure type	Phenotype	Anti-epileptic agent
1	F	2 years, 3 months	1 year	-	-	+	GTCS on VPA	Classical	VPA,
2	F	3 years, 7 months	2 years	-	-	+	GTCS, myoclonic; sporadic absences	Classical	VPA, LTG, CZP
3	M	4 years, 6 months	3 years	-	Paroxysmal hypokinesia	+	-	Non-classical	-
4	M	2 years, 4 months	1 year	-	-	+	-	Non-classical	-
5	F	6 years, 9 months	4 years	-	Paroxysmal hypokinesia	+, IQ 55	-	Non-classical	-
6	M	2 years, 8 months	2 years, 8 months	-	-	+, DQ 56, MI 77	Frequent absences	Classical	VPA, LEV
7	M	5 years	2 years	-	-	+, IQ 40	GTCS	Classical	VPA, OCBZ, Lacosamide
8	M	2 years, 7 months	3 months	-	+	+	Focal seizure	Classical	OCBZ
9	M	12 years, 6 months	1 year	-	-	+	Focal seizure	Classical	VPA, LTG
10	F	6 years	3 years	-	+	+	-	Non-classical	-
11	M	11 years, 9 months	4 years	-	+	+	Generalized seizure	Non-classical	VPA
12	F	3 years,	6 months	-	+	+	-	Non-classical	VPA
13	F	1 years, 8 months	1 year, 1 month	-	-	-	Generalized seizure	Classical	LEV, OCBZ
14	F	5 years, 1 month	2 years, 7 months	-	-	+	Generalized seizure	Classical	LEV, VPA, TPM
15	F	3 years, 9 months	3 years, 1 month	-	-	+	Generalized seizure	Classical	VPA, LTG

F, female; M, male; DD, developmental delay; MD, movement disorder; +, positive; -, negative; GTCS, generalized tonic–clonic seizure; VPA, valproic acid; LTG, lamotrigine; CZP, clonazepam; LEV, levetiracetam; OCBZ, oxcarbazepine; TPM, topiramate.

**Table II:** Laboratory findings and neuroimaging results of patients with GLUT1-DS.

Patients no.	Blood glucose (mmol/l)	CSF glucose (mmol/l)	CSF/blood glucose ratio	Brain MRI	EEG
1	3.8	N/A	N/A	Arachnoid cyst; Bilateral ventricle plump	Epileptiform discharge
2	4.8	1.62	0.33	Normal	Epileptiform discharge
3	5.0	N/A	N/A	Normal	Normal
4	4.6	2.36	0.51	Hypomyelination; Bilateral ventricle plump	Normal
5	3.4	1.34	0.39	Abnormal signals in right frontal lobe	Normal
6	4.6	1.98	0.43	Normal	Background Slowing
7	4.5	1.56	0.35	Left ventricle plump	Background Slowing, δ activity
8	4.37	N/A	N/A	Bilateral ventricle plump	Normal
9	5.4	1.95	0.36	Normal	Epileptiform discharge
10	4.37	N/A	N/A	Left ventricle plump	N/A
11	5.12	2.17	0.42	Bilateral ventricle plump	Epileptiform discharge
12	3.96	1.54	0.39	Normal	Boundary electroencep halogram
13	4.57	2.17	0.47	Several small punctate FLAIR signals in the frontal parietal cortex on both sides	N/A
14	3.9	N/A	N/A	Elevated T2W1 signals in bilateral paraventricular terminal zone	Normal
15	3.96	N/A	N/A	Normal	Background Slowing

N/A, no information available.

**Table III:** Mutation details of patients with GLUT1-DS.

Patients no.	Location in gene	Nucleotide	Amino acid	Source	Type of mutation	Phenotype
1	-	c.997C>T	p.R333W	*De novo*	Missense mutation	Classical
2	-	c.457C>T	p.A153C	*De novo*	Missense mutation	Classical
3	-	c.715dup	p.H239P*2	*De novo*	Frameshift mutation	Non-classical
4	Exon 6	c.695G>A	p.R232H	Father	Missense mutation	Non-classical
5	Exon 4	c.376C>T	p.P126C	*De novo*	Missense mutation	Non-classical
6	Exon 4	c.398G>A	p.C133Y	*De novo*	Missense mutation	Classical
7	IVS 3	c.275+2T>G	-	*De novo*	Splice-site mutation	Classical
8	IVS 5	c.680-11G>A	-	*De novo*	Splice-site mutation	Classical
9	Exon 7	c.884C>T	p.T295M	Father	Missense mutation	Classical
10	Exon 9	c.1199G>A	p.R400H	*De novo*	Missense mutation	Non-classical
11	IVS 5	c.680-1G>T	-	*De novo*	Splice-site mutation	Non-classical
12	Exon 9	c.1077_1090del	p.E359fs*17	*De novo*	Frameshift mutation	Non-classical
13	Exon 8	c.997C>T	p.R333W	Mother	Missense mutation	Classical
14	Exon 4	c.377G>A	p.R126H	*De novo*	Missense mutation	Classical
15	Exon 4	c.464C>T	p.A155V	*De novo*	Missense mutation	Classical

IVS, intervening sequence; DNA mutations were numbered using the reference sequence NM_006516.1.

**Table IV:** Treatment and response of the patients with GLUT1-DS.

			Effect of ketogenic diet on		
Patients no.	Anti-epileptic agent	Clinical features	Movement disorder	Developmental delay	Seizure type
1	VPA	DD, seizure	N/A	+	+
2	VPA, LTG, CZP	DD, seizure	N/A	+	+
3	-	MD, DD	+/-	+	+
4	-	DD	N/A	+	N/A
5	-	MD, DD	+	+	N/A
6	VPA, LEV	DD, seizure	N/A	+	+
7	VPA, OCBZ, Lacosamide	DD, seizure	N/A	N/A	+/-
8	OCBZ	MD, DD, seizure	N/A	N/A	+
9	VPA, LTG	DD, seizure	N/A	N/A	+
10	-	MD, DD	+/-	N/A	N/A
11	VPA	MD, DD, seizure	+/-	+	+
12	VPA	MD, DD	N/A	N/A	N/A
13	LEV, OCBZ	Seizure	N/A	N/A	N/A
14	LEV, VPA, TPM	DD, seizure	N/A	N/A	+
15	VPA, LTG	DD, seizure	N/A	N/A	+/-

The seizure type is shown in Table I. VPA, valproic acid; LTG, lamotrigine; CZP, clonazepam; LEV, levetiracetam; OCBZ, oxcarbazepine; TPM, topiramate; DD, developmental delay; MD, movement disorder; N/A, no information available; Movement disorder: +, total disappearance of movement disorder; +/-, reduction of frequency and/or severity of movement disorder; Developmental delay: +, positive; Seizure type: +, seizure-free; +/-, reduction of seizures.

## Data Availability

The datasets generated and/or analyzed during the current study are not publicly available due to concerns regarding participant/patient anonymity. Requests to access the datasets should be directed to the corresponding author.
